# Cardiac rehabilitation for TAVR patients: mechanisms, current status, and future directions

**DOI:** 10.3389/fcvm.2025.1701764

**Published:** 2025-11-25

**Authors:** Huan Duan, Chuan Zhang, Qi Zhang, Duan Chen, Ling Xue

**Affiliations:** 1 Nursing Department, The First Affiliated Hospital of Chongqing Medical University, Chongqing, China; 2 Department of Cardiovascular Medicine, Cardiovascular Research Center, The First Affiliated Hospital of Chongqing Medical University, Chongqing, China

**Keywords:** aortic stenosis, transcatheter aortic valve replacement, cardiac rehabilitation, multimodal intervention, frailty

## Abstract

Due to the aging population, the prevalence of aortic stenosis continues to rise, transcatheter aortic valve replacement (TAVR) has become an important method for treating severe symptomatic aortic stenosis. Although TAVR significantly improves the survival and symptoms of patients with aortic stenosis, this population is generally characterized by advanced age, frailty, and multiple comorbidities, posing challenges to postoperative functional recovery and quality of life improvement. Cardiac rehabilitation (CR) constitutes a cornerstone of secondary prevention for cardiovascular disease (CVD), and plays a pivotal role in optimizing outcomes for patients undergoing TAVR. This review aims to discuss the mechanistic, current practical evidence, existing challenges, and future directions of CR in TAVR patients.

## 1 Introduction

The prevalence of the most common valvular disease, aortic stenosis (AS), is still increasing, and its prevalence increases with age (1–3). In developed nations, the prevalence of severe aortic stenosis (AS) among individuals aged ≥75 years is 3.4% (4); a 2012–2015 sampling survey of valvular heart disease in China revealed a comparable AS prevalence of 3.4% in the same demographic (5). It is estimated that the number of patients with moderate to severe AS will nearly triple by 2060 (3, 6). TAVR is a safe and effective treatment for inoperable or high-risk patients with severe AS, and its indications have progressively expanded to include low-risk patients (7, 8). Nonetheless, the target population remains frail elderly patients (9). These patients have some postoperative problems, such as cardiac dysfunction, cognitive impairment, and poor recovery of quality of life (10–12). With the continuous growth and popularization of TAVR procedures, the demand for postoperative rehabilitation has surged, making it important to understand the CR outcomes of patients.

CR is a comprehensive cardiovascular disease rehabilitation intervention involving exercise, nutrition, psychology, medication, smoking cessation, and other aspects. Currently, the benefits of CR have been confirmed by numerous studies (13, 14), including reducing mortality, morbidity, and readmission rates, and improving quality of life and physical function, and enhancing metabolic, inflammatory, and molecular biomarkers of risk and resilience (15).

All AS patients may benefit from post-TAVR CR (16). Post-TAVR CR has been proven to be safe and beneficial, it can increase exercise capacity, improve quality of life, and reduce disability, and can reduce patient mortality, morbidity, and readmission rates (17–19). A study by Patel et al. confirmed that AS patients participating in CR had, compared to the control group, a 61% relative risk reduction in 1-year mortality, a 4.2% absolute risk reduction, and a 34% relative reduction in readmission risk (20). Furthermore, CR may also positively impact patients’ return to work and is cost-effective (21). Its implementation is recommended by clinical practice guidelines (22). The 2020 updated position statement from the European Association of Preventive Cardiology on secondary prevention and rehabilitation states that all patients after aortic valve replacement should receive a CR program (23). The 2020 ESC guidelines on sports cardiology and exercise in patients with cardiovascular disease suggest that patients with asymptomatic mild AS can participate in all sports, while patients with symptomatic AS can consider light exercise that does not provoke symptoms for general health benefits (24). A Chinese expert consensus proposes that early exercise rehabilitation should be initiated as soon as possible after TAVR, provided patient safety is ensured (25).

However, in the real world, the referral rate for CR in TAVR patients is low (19, 26). A U.S. study found that only 39.8% (4027/10,124) of TAVR patients participated in CR (19). The CR referral rate in China is 12.4%, which is lower than the global average (34.4%) (27). The reasons for the low CR referral rate are multifaceted, including lack of or inefficient referral processes, limited health insurance coverage, scheduling conflicts, and inconvenience or long distances leading to excessive time and transportation costs for participating in CR (28, 29). A qualitative study further revealed the barriers and facilitators affecting CR acceptance in AS patients over 80 years old, primarily involving Perceptions and Understanding, Delivery and Accessibility, Perceived Impact of Exercise and Health and Life Changes, and Transportation (30). In conclusion, the risk of cardiovascular events remains high after TAVR, and CR can definitively improve patient prognosis and quality of life. However, the proportion of patients receiving CR in the real world is low. Future research could explore more accessible CR programs to address the multiple barriers TAVR patients face in participating in CR.

This review will discuss the pathophysiological mechanisms of CR in TAVR patients, comprehensive interventions covering the four core dimensions of exercise, nutrition, psychology, and risk factors, and then delve into the three major aspects of individualized clinical practice for complications, frailty, and digital health.

We conducted a systematic search of the literature to examine the existing evidence on CR after TAVR. Our search strategy combined keywords and Medical Subject Headings, including “transcatheter aortic valve replacement”, “TAVR”, “transcatheter aortic valve implantation”, “TAVI”, “cardiac rehabilitation”, “cardiovascular rehabilitation”, etc. These terms were combined using Boolean operators (AND, OR). Due to the limited number of studies identified in the initial search, we used a snowballing method to manually review the reference lists of relevant articles. The primary databases searched were PubMed, Web of Science, and Science Direct. The inclusion criteria included randomized controlled trials, cohort studies, retrospective studies, systematic reviews, and expert consensus publications; case reports were excluded.

## 2 The pathophysiological mechanisms of CR in TAVR patients

Clinical risk factors for the development of AS include advanced age, hypertension, hyperlipidemia, smoking, and diabetes (31), characterized by progressive fibrotic/calcific narrowing of the aortic valve, a process that is highly complex, involving lipoprotein deposition, chronic inflammation, osteogenic transformation of valvular interstitial cells, and active leaflet calcification (4). Progressive aortic valve stenosis leads to an increase in left ventricular (LV) afterload (32). The LV must continually overcome the resistance generated by the stenotic valve to maintain cardiac output, which over time leads to compensatory hypertrophy of LV cardiomyocytes, and increased LV wall thickness and mass (6, 33, 34). In the early stages of the disease, the body maintains a near-normal cardiac output through a delicate balance between left ventricular preload and afterload, with increased preload to provide adequate stroke volume, and increased afterload to maintain perfusion pressure. Subtle factors affecting preload or afterload may precipitate acute decompensation (35). Without intervention, disease progression leads to irreversible myocardial damage, diastolic/systolic dysfunction (6), decreased left ventricular ejection fraction (LVEF) (36), reduced coronary flow reserve (CFR) (37), and increased myocardial oxygen consumption (38), all leading to limited cardiac output.

A reduction in cardiac output leads to prioritized redistribution of blood flow to vital organs such as the brain, resulting in insufficient blood perfusion to skeletal muscles, increased local vascular resistance, and reduced shear stress and chronic inflammation, which inhibits nitric oxide (NO) release and consequently causes vascular endothelial dysfunction (39, 40).

**Figure 1:**
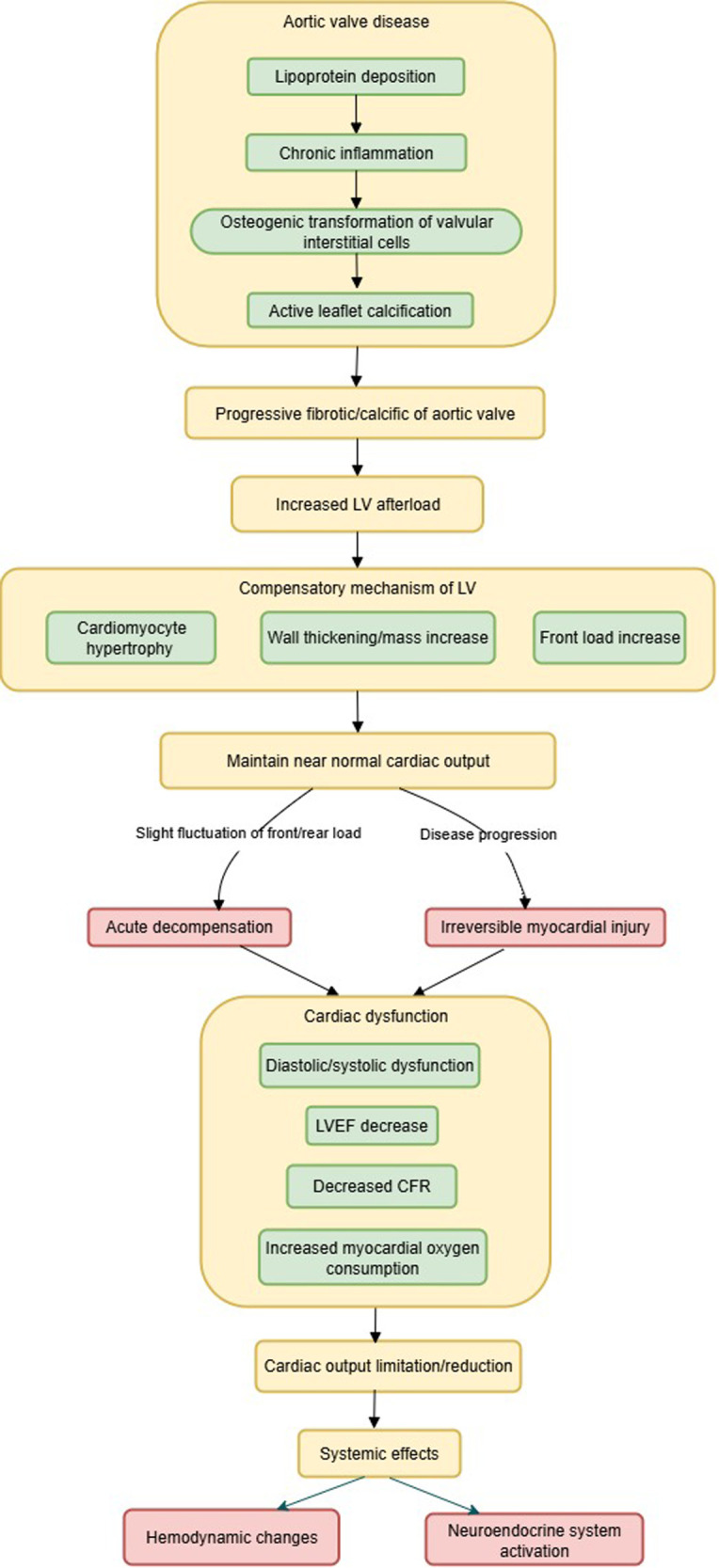
The pathophysiological mechanism of AS.

Furthermore, AS activates multiple neuroendocrine systems, such as activation of the sympathetic nervous system (SNS) (41), the renin-angiotensin-aldosterone system (RAAS) (42), the natriuretic peptide system (BNP/NT-proBNP) (43), and inflammatory pathways (44). Research has confirmed that neurohormonal activity is reduced in patients after TAVR, resulting in significant clinical benefits (45). The pathophysiological mechanism of AS is shown in Figure 1.

Currently, no effective pharmacotherapy exists to alter the natural history of AS progression (32). TAVR can directly reverse AS, resulting in a significant reduction or even elimination of the aortic transvalvular pressure gradient, causing an abrupt decrease in LV afterload and a marked reduction in myocardial oxygen consumption. In AS patients with preserved systolic function, stroke volume and cardiac output usually increase rapidly. This improves systemic tissue perfusion, thereby alleviating patient symptoms such as dyspnea, fatigue, and angina.

CR cannot directly relieve valvular stenosis like TAVR does, but it plays an irreplaceable role in ensuring the smooth progress of TAVR and maintaining the surgical outcomes. The mechanisms of CR are multifaceted, interactive, and comprehensive, with its core function being to improve hemodynamics, reverse cardiac remodeling, and enhance overall functional capacity through regular exercise. Studies indicate that exercise training can improve ventilatory function, endothelial function, and enhance skeletal muscle metabolism, and reduce levels of inflammatory cytokines (e.g., tumor necrosis factor-α, and interleukin-6), exerting positive effects on peak VO2, autonomic nervous system function, central hemodynamic function, peripheral vascular and muscle function, and exercise capacity (15, 46–48). It must be emphasized that exercise training should be regular and incorporate aerobic exercise.

TAVR patients often experience anxiety, depression, and concerns about their prognosis. Depressive status is associated with poor nutritional choices, limited exercise, smoking, and reduced medication adherence, which may exacerbate heart disease (49). CR influences rehabilitation outcomes by providing psychological counseling, patient education, and behavioral interventions to help patients reduce psychological stress, improve nutrition, quit smoking, control blood pressure, blood glucose, and lipids, and enhance medication adherence.

## 3 Core components of integrated CR after TAVR: exercise, nutrition, psychological support, and risk factor management

CR is a multidimensional and personalized rehabilitation process, and the synergistic effect of all aspects is crucial for patient recovery and long-term health. In post-TAVR management, multimodal CR that integrates exercise, nutrition, psychological support, and risk factor control can provide comprehensive recovery support for patients. By improving patients’ psychological burden and nutritional status, it can also indirectly enhance their participation and completion rates of the rehabilitation program, thus forming a virtuous cycle.

A multicenter prospective cohort study implemented a three-week standardized multi-component inpatient CR for 136 elderly patients after TAVR, including patient education, personalized exercise training, dietary counseling, psychological support, and risk factor management. This study found that patients’ physical capacity, quality of life, anxiety, and frailty were significantly improved (50). A nurse-led multidimensional CR program (including exercise, medication management, nutritional guidance, psychological support, sleep management, health education, and smoking cessation assistance) also showed significant benefits for frailty status, 6MWT, walking speed, grip strength, etc. in patients with unstable angina (UA) undergoing percutaneous coronary intervention (PCI) (51). Takashi et al. found that comprehensive CR supervised by a multidisciplinary rehabilitation team can significantly reduce major adverse cardiovascular events in acute myocardial infarction (AMI) patients undergoing PCI (52). Moreover, the benefits of multimodal CR for heart failure and atrial fibrillation patients have also been confirmed (53, 54).

Currently, there are few large-scale randomized controlled trials related to CR after TAVR, Exercise-based CR is a key intervention for cardiovascular diseases (CVD), with most studies focusing on the exercise rehabilitation aspect. There is a need to increase exploration in areas such as nutrition and psychological support.

### 3.1 Exercise rehabilitation

Studies show that 93.33% of TAVR patients lack regular physical activity and have low adherence (55). Sedentary behavior increases the risk of mortality and functional decline after TAVR (56), while moderate-to-vigorous physical activity and participation in CR can improve survival rates in patients after heart valve surgery [OR,1.52, (95% CI 1.03–2.24)] (57). Exercise-based CR programs after TAVR have been proven safe and can significantly improve physical capacity and health-related quality of life (9), but this may be independent of program duration (58). Currently, there is no unified protocol for post-TAVR exercise rehabilitation, with significant variations between study protocols, as shown in Table 1. Future efforts should further develop intervention strategies to enhance patients’ motivation and ability for physical activity and provide appropriate triggers.

**Table 1:** Overview of clinical research on exercise rehabilitation.

Author/year of publication/country	Type of study	Number of patients	Exercise program	Key findings
Song et al. 2024/China (123)	RCT	Exercise training (ET) group (*n* = 30) Control group (*n* = 30)	Progressive aerobic training. Patients exercised for a total of 40 min/d, consisting of 5 min warm-up, 30 min individual exercise prescription, and 5 min rest period. The exercise frequency was 5 days per week for 12 weeks. Oxygen saturation, heart rate, and blood pressure were monitored during CR exercise sessions.	Improved cardio pulmonary function, exercise tolerance, and quality of life.
Xu et al. 2023/China (124)	RCT	Inspiratory muscle training (IMT) + CR group (*n* = 48) CR group (*n* = 48)	Sports training includes bed activity training, “sitting and standing” transfer training and aerobic walking training. Exercise for 30 min each time, once a day, 3 to 5 days a week, lasting for the whole hospitalization period (average 11 days). The IMT course is divided into three stages. First, the subjects completed 5 min of breathing control training and chest expansion and stretching exercises in the warm-up phase. Then, with the help of a portable spirometer, participants completed 10 min of moderate to high resistance inspiratory training. Finally, five minutes of breathing training was performed in the recovery phase. All participants performed supervised IMT with a portable spirometer. The intervention group received supervised IMT treatment, and the control group received IMT treatment with the sham device.	Improving exercise endurance, and pulmonary ventilation function, increasing inspiratory muscle strength, and shortening the length of hospital stay.
Hu et al.2023/China (125)	RCT	Moderate intensity continuous training (MICT *n* = 33) Control group (*n* = 33)	MICT was scheduled 3 times per week for 3 months. Every session Included warm-up (<50% target intensity for 2 min, then gradually increasing the load 1 to 10 W/min up to 100% target intensity for 5–10 min), exercise phase (100% target intensity starting with 20 min and gradually lengthening up to 45 min in the 1 month or maintaining for 45 min in the following 2 months), cool down with gradual reduction of load within 3 min.	The MICT group had more significant changes in peak VO_2_, 6MWT, and LDL cholesterol. MICT had a positive effect on the cardiopulmonary function and physical capacity of patients after TAVR.
Brocki et al. 2023/Denmark (113)	Single center feasibility study	13 patients (8 patients did not complete the study)	It is initiated one week after TAVR and lasts for a total of 12 weeks, with the first 8 weeks being supervised training. It adopts a combined mode of aerobic exercise and strength training, conducted twice a week, and each training session lasts 30–45 min. In addition, patients are also instructed to take a 30-minute walk at a moderate intensity level every day.	6MWT and grip strength were significantly improved, but gait speed, 30 s sitting and standing test and QOL were not significantly changed, and the participation rate and retention rate of patients were low.
Vitez et al. 2023/Slovenia (126)	Prospective, single center RCT	Center-based exercise training (*n* = 20) Unsupervised home-based exercise (*n* = 19)	An 8–12 week outpatient exercise training program with two visits per week (16–24 visits in total). Each training session consists of a 10-minute warm-up, moderate-intensity endurance training, low-to-moderate-intensity resistance training, balance training, and a 10-minute cool-down. Among these components, the moderate-intensity endurance training involves 20 min of cycling [starting at an initial intensity of 40% of peak oxygen consumption (VO₂ peak), followed by a gradual increase to the target intensity—75% of VO₂ peak—and a gradual extension of the training duration to 40 min]; the low-to-moderate-intensity resistance training is conducted using free weights and resistance bands. Control group: a 30 min information meeting was held with the physiotherapist, and it is recommended to exercise regularly for at least 150 min every week.	Both supervised and unsupervised exercise training can improve exercise capacity and vascular function in patients after TAVR, with supervised exercise training showing a significantly greater improvement in vascular function.
Lindman et al. 2021/America (127)	RCT	Intervention group(*n* = 25) Control group(*n* = 25)	Intervention group: An activity monitor was used to display daily step count, time, distance traveled, heart rate, and other metrics. Participants were assigned a tailored daily step goal; when they achieved this daily step goal, they received a notification on the activity monitor and a vibration each hour at ten minutes to the hour to encourage at least 250 steps/h. Participants in the intervention group were also required to perform daily resistance exercises, including 5–10 chair sit-to-stand exercises (to strengthen the lower limbs), 5–10 chair push-ups (to strengthen the upper limbs), and 10 stress ball squeezes (to strengthen grip strength). The training cycle was performed 1 to 5 times daily, completed on 6 days per week over six weeks. Daily training reminders were sent via iPad, and participants were required to answer a questionnaire on whether they completed the training and the number of cycles completed each day. Control group: The activity monitor only displayed the time and gave no reminders or feedback and no instructions, reminders, or queries about exercise were given.	The intervention group had high compliance and was associated with more daily physical activity. Exercise intervention did not improve the physical function or quality of life of the entire study cohort.
Tamulevičiūtė-Prascienė et al. 2021/Lithuania (128)	RCT	Intervention group (IG *n* = 60) Control group (CG *n* = 56)	Both IG and CG are required to complete a 20 days usual exercise training course. (1) continuous endurance training on cycle ergometers (6 sessions a week). Every session included warm-up (<50% target intensity 2 min, gradually increasing load 1–10 W/min up to target intensity within 5–10 min); exercise phase, 100% of the target intensity, starting with >5 min and gradually lengthening up to 30 min; cool-down with gradual reduction of the load within 3 min; (2) aerobic dynamic gymnastics in sitting and/or standing position (30 min, 5 days/week); (3) respiratory muscle training (7 days/week, for 15 min). The intervention group received additional resistance and balance training, and were followed up for 12 weeks. Resistance training starts at low intensity and gradually increases to moderate intensity for 3 groups, with a 3-minute break between groups. The balance training included exercises to improve static and dynamic balance ability. It was performed on 2–3 days/week for 10–15 min.	Exercise-based inpatient CR improves functional capacity, physical performance, exercise capacity and muscular strength in patients after valve surgery or intervention in the short and medium terms. The additional specially tailored resistance/balance training was accepted and tolerated in the patient cohort.

### 3.2 Dietary/nutritional support

Dietary advice plays an important role in CR programs and is associated with improved metabolic and cardiovascular outcomes (59). t is reported that adherence to a Mediterranean diet can reduce cardiovascular events by 30% (59, 60). A study by Schwaab et al. found that CR can improve low-density lipoprotein cholesterol (LDL-C) levels in patients with myocardial infarction (MI), with the proportion of patients achieving an LDL-C concentration below 70 mg/dL or a reduction ≥50% increasing from only 2% at baseline to 42% after an average of 22 days of treatment (61). TAVR patients are often very frail (62); conducting nutritional risk assessments and providing personalized dietary advice are crucial for their early recovery (63). However, it is difficult for frail patients to follow dietary advice in actual practice.

#### 3.2.1 Effect of malnutrition on patients with TAVR

Malnutrition is a common yet often overlooked problem in TAVR patients, and its presence predicts a poor prognosis, particularly being significantly associated with increased mortality (64–66). A prospective multicenter cohort study of 1,158 patients undergoing aortic valve replacement (TAVR or surgical aortic valve replacement, SAVR) showed that compared to patients with normal nutritional status, older adults with signs and symptoms of malnutrition had a nearly threefold increase in one-year postoperative mortality (28% vs. 10%, *P* < 0.001) (67). This indicates that nutritional status is an important indicator for predicting one-year all-cause mortality in TAVR patients (68).

#### 3.2.2 Assessment of nutritional risk in patients with TAVR

Nutritional status assessment is challenging, and traditional indicators such as body weight are often unreliable (65, 67). Multiple studies using different nutritional screening tools have revealed a high risk of malnutrition in this population. Mini Nutritional Assessment-Short Form (MNA-SF): Goldfarb et al. screened 1,158 patients (727 TAVR and 431 SAVR) for nutritional risk and found that 32.8% were at risk of malnutrition (67). Geriatric Nutritional Risk Index (GNRI): Data from a single-center prospective study of 433 TAVR patients showed that 61.4% of the patients were at nutritional risk before TAVR (mild 17.6%, moderate 40.6%, severe 3.2%) (69).The study also found that patients whose malnutrition improved after TAVR had better survival than those with persistent malnutrition (69). Another study involving 953 TAVR patients found that a low GNRI (<98, prevalence 35.2%) was associated with an increased risk of all-cause mortality (70).

Composite indices [the Controlling Nutritional Status score (CONUT), GNRI, Prognostic Nutritional Index (PNI)]: Ishizu et al. found in a study of 1,040 Japanese high-risk TAVR patients that the proportions of moderate/severe malnutrition were: CONUT 16.6%, GNRI 60.5%, PNI 13.8% (65). A study by Li et al. on 536 Chinese AS patients also reported similar discrepancies: CONUT 14.4%, NRI 44.4%, PNI 10.6% (71). Collectively, it is evident that NRI/GNRI detects a higher proportion of nutritional risk, while PNI shows the opposite trend.

MNA-SF, CONUT, NRI/GNRI, and PNI have been proven to predict the prognosis of TAVR patients (64, 66, 67, 72). Among them, MNA-SF involves patient self-reported nutritional status, CONUT assesses serum albumin levels, total cholesterol levels, and total lymphocyte count (64), NRI/GNRI assesses serum albumin and body weight/body mass index (66, 73), and PNI assesses serum albumin and total lymphocyte count (72). It is noteworthy that CONUT, NRI/GNRI, and PNI all measure the objective indicator of serum albumin, possibly suggesting that its level is associated with the prognosis of TAVR patients (74–76). Interestingly, two small studies comparing the value of GNRI, PNI, and CONUT in predicting one-year mortality after TAVR yielded conflicting results (one found CONUT/PNI superior, the other found GNRI superior) (77, 78).

Novel indicators are also being explored; the Triglycerides × Total Cholesterol × Body Mass Index (TCBI) is a simple nutritional scoring calculation method that has been validated in CVD patients (79, 80). Sudo et al. found that a low TCBI was associated with signs of right heart overload and an increased risk of 3-year mortality. Its addition to the EuroSCORE II enhanced the predictive value for all-cause mortality (81). A study utilizing the Malnutrition Universal Screening Tool (MUST) and 3-day food diaries administered by dietitians and nurses found that 8 patients (22%) were at medium risk and 3 patients (8%) were at high risk; 6 patients had a nutritional intake of <50% of their calculated requirements, and 3 of these patients required early nutritional support (62).

In summary, the prevalence of malnutrition in TAVR patients varies depending on the assessment tool used, but regardless of which nutritional indicator is used, the deterioration of malnutrition is associated with higher all-cause mortality (71). Future research is needed to clarify the clinical value of different nutritional assessment indicators in predicting adverse outcomes in TAVR patients.

#### 3.2.3 Nutritional support for TAVR patients

Given that malnutrition can be reversed through nutritional intervention, early nutritional risk assessment before TAVR is crucial, as it helps clinicians identify patients who may benefit from nutritional support to improve the prognosis of TAVR patients. Therefore, routine screening of patients’ nutritional status should be performed before TAVR, and interventions should be provided when appropriate to improve outcomes (68). The PERFORM-TAVR trial, a multicenter randomized clinical trial with a parallel-group design exploring the effects of nutritional intervention, concluded on December 1, 2022. The trial aimed to assess whether protein supplementation combined with exercise could improve physical frailty in elderly TAVR patients (defined as an SPPB ≤8 or an SF36-PF ≤55). Patients in the intervention group received a protein-rich nutritional supplement twice daily from 4 weeks before TAVR until 12 weeks after discharge; the study results have not yet been published. A substantial body of research indicates that nutritional support is effective in improving the prognosis of patients with CVD (59, 82, 83). However, no other studies have specifically examined the independent effect of nutritional support on the rehabilitation of TAVR patients. Therefore, the results of the PERFORM-TAVR trial are highly anticipated, and it is hoped that more researchers will focus on improving the nutritional status of TAVR patients.

### 3.3 Psychological support

Compared to quality of life, the psychological health status of TAVR patients receives less attention, however, preoperative anxiety and depression are quite prevalent among TAVR patients (84). A systematic review found that the prevalence of depression and anxiety in TAVR patients was 15%–30% and 25%–30% (85). Depression is associated with mortality; the presence of depressive symptoms before TAVR is associated with a higher risk of short- and medium-term mortality (86). If depression persists postoperatively, the mortality risk is highest (86). Moreover, depression and cognitive impairment have an additive effect in predicting mortality risk after TAVR (Depression: HR,1.45, [95% CI, 1.13–1.86], *P* < 0.01; Cognitive Dysfunction(CD): HR,1.27, [95% CI, 1.02–1.59], *P* = 0.04; Depression and CD: HR,2.06, [95% CI, 1.44–2.96], *P* < 0.01) (87).

The effectiveness of psychological interventions for TAVR patients shows inconsistent results. A study found that CR significantly improved depressive symptoms in TAVR patients (PHQ-9 score decreased from 4.3 to 2.3, *P* < 0.01), but failed to improve anxiety symptoms (GAD-7 score decreased from 3.4 to 2.3, *P* = 0.25) (26). A RCT confirmed that CR after cardiac valve surgery improved physical capacity but did not improve mental health (88). A psychological education program specifically for cardiac valve surgery patients, implemented within CR, also failed to improve their physical and mental health status at 12 or 24 months (18). Another study reported no between-group differences in Hospital Anxiety and Depression Scale (HADS) scores at different time points after discharge (30 days, 6 months, 12 months), regardless of whether inpatient CR was arranged (16). Interestingly, one RCT observed spontaneous improvement in depression and anxiety scores in TAVR patients at 1 month postoperatively, and brief cognitive behavioral therapy (CBT) showed no additional advantage (84). However, this does not seem to be a universal phenomenon, as a large-sample study found that even after excluding patients with recorded anxiety or depression within 6 months before TAVR, the cumulative incidence of new-onset anxiety or depression 1 year after TAVR remained as high as 19% (89).

Anxiety and depressive symptoms may resolve spontaneously in some TAVR patients, and some studies have questioned the effectiveness of CR in improving the mental health of TAVR patients. However, mental health issues in TAVR patients are real and associated with poor prognosis. Future research aimed at optimizing post-TAVR mental health rehabilitation should more precisely focus on patient populations with elevated baseline psychological symptoms (90), and delve deeper into exploring more effective targeted interventions.

### 3.4 Risk factor management

TAVR significantly improves patients’ symptoms and long-term prognosis, but patients may still face various challenges postoperatively, such as complications, lack of rehabilitation knowledge, and long-term durability issues of bioprosthetic valves, etc. The effectiveness of CR is often measured by the reduction of CVD risk factors (91), CR manages risk factors through systematic patient education and behavioral interventions, including promoting smoking cessation, effectively controlling blood pressure, blood glucose, lipids, weight management, and improving medication adherence, thereby optimizing long-term prognosis, addressing patients’ knowledge gaps in rehabilitation, enhancing their postoperative self-management skills, and preparing them for long-term challenges such as valve durability.

Studies have found that cardiovascular risk factors such as smoking, hypercholesterolemia, and elevated serum creatinine and calcium levels accelerate disease progression in AS patients (92). The ACC/AHA guidelines for the management of patients with valvular heart disease clearly state that smoking is associated with adverse outcomes after TAVR (7). Smoking cessation is the most important modifiable cardiac risk factor and is recognized as a key secondary prevention strategy. Furthermore, conventional cardiovascular risk factors such as hypertension, diabetes, and dyslipidemia have a strong, independent, and graded association with the incidence of severe AS in the elderly, accounting for about one-third of its attributable risk (93). Studies have also found that smoking, diabetes, high cholesterol, and triglyceride levels are associated with accelerated bioprosthetic valve degeneration (94). In a multivariate regression model, gender, smoking, diabetes, and cholesterol levels were significant risk factors predicting the need for reoperation (94). Patients without these risk factors required reoperation after an average of 9.25 ± 0.88 years, which was significantly shortened to 4.05 ± 0.43 years in patients with 2 or 3 risk factors (*P* = .0002) (94). Therefore, for TAVR patients with these risk factors, the CR team must emphasize and guide strict control of these high-risk factors, which is crucial for delaying bioprosthetic valve degeneration and avoiding early reoperation.

Pharmacological management is also a crucial component of CR, as the majority of patients post-TAVR require medication to prevent valve thrombosis and thromboembolic events. The European Society of Cardiology/European Association for Cardio-Thoracic Surgery and the ACC/AHA have issued guidelines for post-TAVR antithrombotic therapy (7, 95). However, due to a lack of high-quality evidence, specific antithrombotic regimens remain controversial (8). Pharmacologic regimens are individualized and adjusted by cardiologists based on the latest guidelines and patient-specific circumstances. Patients should strictly adhere to prescribed medications postoperatively and attend regular follow-up visits.

## 4 Clinical challenges and countermeasures in post-TAVR rehabilitation: complications, frailty, and individualized practice of digital health

### 4.1 Rehabilitation management of complications after TAVR

TAVR is a minimally invasive procedure, but patients have numerous and frequent postoperative complications (96). These complications undoubtedly increase the difficulty of CR management in TAVR patients and may even hinder the implementation of CR. Therefore,CR programs need to be individually adjusted based on the patient's personalized complication risks.

For patients with moderate/severe paravalvular aortic regurgitation (97), close monitoring for heart failure symptoms such as dyspnea and fatigue during rehabilitation is essential. The exercise rehabilitation should be conservative, avoiding resistance training that may cause drastic blood pressure fluctuations, and symptom-limited exercise can be performed. If severe vascular complications and bleeding occur (98), the timing of rehabilitation should be appropriately delayed. For patients via the femoral artery approach, exercise rehabilitation of the lower limb muscles can be initiated after the puncture site has healed. For patients with disabling stroke (99), the focus of CR should be comprehensive neuro-cardiac rehabilitation addressing cardiac, cerebral, and limb functions. The rehabilitation program should focus on training muscle strength, balance, coordination, and activities of daily living, and the multidisciplinary team should include neurologists. For patients with acute kidney injury (100), exercise intensity should be appropriately reduced. Rehabilitation mandates rigorous monitoring of fluid balance and electrolyte levels, and strenuous activities that may induce dehydration should be avoided prior to full renal function recovery. For patients with infective endocarditis (101), rehabilitation should commence gradually after the infection is controlled and safety is assessed by a multidisciplinary team, while simultaneously enhancing nutritional support for the patient. For patients who develop high-grade atrioventricular block (102), close monitoring of ECG evolution is required during rehabilitation before pacemaker implantation. During exercise rehabilitation, vigilance for bradycardia or even syncope is necessary.

It is reported that permanent pacemaker implantation (PPI) is the most common complication of TAVR, with a post-TAVR PPI incidence rate ranging between 2% and 51%. In the foreseeable future, a significant reduction in this type of complication after TAVR is unlikely (102). CR for this patient population faces unique challenges. Cardiac implantable electronic devices often impose a specific psychological burden on patients, such as concerns about limitations in daily life and device function (103), which may reduce their willingness to participate in rehabilitation and their quality of life. Rehabilitation personnel should provide timely activity guidance, proactively assess the patient's psychological state, and offer targeted psychological counseling. Infection after PPI and lead dysfunction are uncommon but represent a serious complication. Transvenous lead extraction is the gold standard for treating such complications (104). Rehabilitation personnel need to teach patients to promptly recognize signs of infection and inform them of the importance of postoperative antibiotic use.

### 4.2 Rehabilitation management of frailty after TAVR

TAVR patients are often elderly with numerous comorbidities, and the incidence of postoperative frailty varies depending on the assessment tool, the FRAILTY-AVR study reported an incidence ranging from 26% to 68% (105). Frailty is a significant risk factor for postoperative disability and mortality (95), making frailty in TAVR patients an important issue that cannot be ignored. Although CR has been proven to effectively improve frailty and health outcomes (106), particularly for frail patients with physical deconditioning, recent surgery, high psychological burden of disease, or cognitive limitations (15), the key to maximizing its benefits lies in integrating standardized frailty assessments into the CR protocol and formulating individualized rehabilitation strategies based on the results. The use of validated, standardized assessment tools is recommended in both clinical practice and research. In CR for TAVR patients, screening with standardized assessment tools before and during rehabilitation enables the objective quantification of frailty severity, providing a crucial basis for setting rehabilitation intensity. Currently, there is no consensus on the optimal method for measuring frailty. The Fried Frailty Phenotype is the most commonly used multidimensional measure (107), while the Clinical Frailty Scale is a simpler unidimensional measure. Both tools have been validated in TAVR patients for effectively predicting mortality (108, 109).The core of identifying frailty lies in using the assessment results to precisely guide CR practice. For patients with severe frailty, the goals are to maintain existing function, prevent disability, and reduce complications. The rehabilitation intensity in the initial phase needs to be extremely conservative, focusing on low-intensity, short-duration activities. The progression cycle should be longer than that for patients with mild/moderate frailty. CR for frail TAVR patients should not be limited to exercise rehabilitation alone but should adopt a multimodal, comprehensive CR approach. Post-TAVR frailty is closely associated with the occurrence of postoperative complications and poor recovery. When formulating a CR plan, it is essential to consider their frailty status alongside specific postoperative complication risks. Some scholars suggest that patients who participate in prehabilitation may exhibit higher adherence to postoperative CR (110), and it can also reduce the risk of postoperative complications, shorten hospital stays, promote recovery, and improve quality of life in patients undergoing major surgery (111). Therefore, rehabilitation can be initiated preoperatively for AS patients to enhance the physical and psychological readiness of frail patients, facilitating the smooth implementation of postoperative CR. It is worth mentioning that tele-rehabilitation offers distinct advantages for discharge CR in frail patients.

### 4.3 Application of digital CR in TAVR patients

Currently, digital health technologies are significantly enhancing the personalization, convenience, and monitoring level of CR programs. Research confirms that digital CR is as effective as, or even superior to, traditional CR in improving patient outcomes (28). Digital CR can better promote and sustain patients’ health behaviors, and is conducive to addressing the underutilization of CR to promote health equity (112). Several studies have utilized remote monitoring technologies such as the internet, wearable devices, and mobile applications to effectively address issues like geographical barriers to CR participation and low rehabilitation adherence in TAVR patients (113–116). Moreover, digital CR demonstrates effectiveness comparable to hospital-based CR programs in reducing the incidence of postoperative complications, readmission rates, and mortality (114, 117, 118).

Kohei et al. used remote monitoring applications and wireless ECG transmitters to conduct remote cardiac rehabilitation (RCR) management for TAVR patients and found that the patients’ peak VO_2_ and 6MWT values were significantly greater than before the intervention (114). A U.S. research team developed the multimodal Corrie Health digital platform, which utilizes smartphones, smartwatches, and wireless blood pressure monitors to assist 1,064 patients recovering from AMI in self-management. The intervention group using Corrie had a 52% lower risk of readmission within 30 days after discharge compared to the control group [HR, 0.48 (95% CI, 0.26–0.88), *P* = 0.018] (119). The research team is integrating the multimodal Corrie Health digital platform with CR to explore the efficacy and safety of digital CR for patients after AMI, coronary artery bypass graft surgery, PCI, cardiac valve surgery, or TAVR (120). Currently, the application of digital CR in TAVR patients is still insufficient, while its application in patients with coronary heart disease is relatively more extensive (51, 121, 122). Future exploration by more scholars in this field is anticipated.

## 5 Existing challenges and future directions

Although the benefits of CR for TAVR patients have been confirmed, its full potential has not been fully realized due to a series of challenges. Action must be taken to fully utilize CR to optimize the long-term outcomes of this growing and complex patient population.

Post-TAVR CR faces many challenges. Firstly, there is a lack of high-quality evidence and standardization. There are few large-scale randomized controlled trials focusing on CR after TAVR. This evidence gap leads to a lack of standardized, universally accepted CR protocols. In terms of exercise rehabilitation, current protocols show significant variations in intensity, frequency, and duration, hindering comparative effectiveness research and consistent clinical implementation. Secondly, CR participation rates and adherence are suboptimal, resulting from multiple barriers. Third, the high prevalence of frailty and multiple comorbidities complicates the implementation of CR. Additionally, patients often have concomitant malnutrition and psychological distress, which are frequently underemphasized in traditional exercise-centric CR models. Fourth, the management of postoperative complications. The high incidence and variety of post-TAVR complications necessitate careful, conservative adjustments to CR protocols, complicating the implementation of a “one-size-fits-all” approach and requiring an individualized strategy.

Future strategies could adopt personalized and multimodal CR, not just focusing on exercise rehabilitation. CR protocols should be comprehensive and personalized, integrating tailored exercise prescriptions with structured nutritional support and psychological interventions. The systematic use of validated assessments for frailty, nutrition, and mental health is crucial for designing these individualized protocols. Furthermore, robust clinical trials are needed to establish standardized protocols and confirm the efficacy of novel interventions like prehabilitation and digital CR for TAVR patients. The foundation for all these efforts must be strong multidisciplinary team support, ensuring seamless collaboration among cardiologists, rehabilitation specialists, nutritionists, psychologists, and nursing specialists to provide holistic care.

In conclusion, although post-TAVR CR still faces significant challenges, it also implies abundant opportunities. The future lies in embracing personalized, multimodal rehabilitation by leveraging the power of prehabilitation and digital technology, and promoting strong multidisciplinary collaboration. Through these synergistic efforts, CR can transform from an underutilized intervention into a rehabilitation modality that provides high-quality rehabilitation care for all TAVR patients.
